# Future climate change is predicted to affect the microbiome and condition of habitat-forming kelp

**DOI:** 10.1098/rspb.2018.1887

**Published:** 2019-02-06

**Authors:** Zhiguang Qiu, Melinda A. Coleman, Euan Provost, Alexandra H. Campbell, Brendan P. Kelaher, Steven J. Dalton, Torsten Thomas, Peter D. Steinberg, Ezequiel M. Marzinelli

**Affiliations:** 1 Centre for Marine Bio-Innovation, School of Biological, Earth and Environmental Sciences, University of New South Wales, Sydney, New South Wales 2052, Australia; 2 Department of Primary Industries, NSW Fisheries, PO Box 4321, Coffs Harbour, New South Wales 2450, Australia; 3 National Marine Science Centre, Southern Cross University, Coffs Harbour, New South Wales 2450, Australia; 4 GeneCology Research Centre, University of the Sunshine Coast, Queensland 4556, Australia; 5 School of Biological Sciences, University of Queensland, St Lucia, Queensland 4072, Australia; 6 Sydney Institute of Marine Science, 19 Chowder Bay Road, Mosman, New South Wales 2088, Australia; 7 Singapore Centre for Environmental Life Sciences Engineering, Nanyang Technological University, 60 Nanyang Drive, SBS-01N-27, Singapore 637551, Republic of Singapore; 8 School of Life and Environmental Sciences, Coastal and Marine Ecosystems, University of Sydney, Sydney, New South Wales 2006, Australia

**Keywords:** acidification, bacteria, disease, *Ecklonia radiata*, holobiont, temperature

## Abstract

Climate change is driving global declines of marine habitat-forming species through physiological effects and through changes to ecological interactions, with projected trajectories for ocean warming and acidification likely to exacerbate such impacts in coming decades. Interactions between habitat-formers and their microbiomes are fundamental for host functioning and resilience, but how such relationships will change in future conditions is largely unknown. We investigated independent and interactive effects of warming and acidification on a large brown seaweed, the kelp *Ecklonia radiata*, and its associated microbiome in experimental mesocosms. Microbial communities were affected by warming and, during the first week, by acidification. During the second week, kelp developed disease-like symptoms previously observed in the field. The tissue of some kelp blistered, bleached and eventually degraded, particularly under the acidification treatments, affecting photosynthetic efficiency. Microbial communities differed between blistered and healthy kelp for all treatments, except for those under future conditions of warming and acidification, which after two weeks resembled assemblages associated with healthy hosts. This indicates that changes in the microbiome were not easily predictable as the severity of future climate scenarios increased. Future ocean conditions can change kelp microbiomes and may lead to host disease, with potentially cascading impacts on associated ecosystems.

## 1. Introduction

Climatic change is affecting biodiversity at a global scale [1]. In the marine realm, ocean warming and acidification are driving regime shifts whereby dominant habitat-forming species, such as corals and large seaweeds, are being replaced by less complex and less productive habitats, affecting biodiversity [2–4]. Understanding the mechanisms that underpin these climate-driven shifts is critical to properly manage and conserve marine ecosystems [5].

Ocean warming and acidification can have direct physiological effects on habitat-forming species, negatively affecting their performance and survival [6] (but see [7]). Often, marine regime shifts are driven by indirect effects of these stressors via changes in species' interactions [8–10]. However, studies typically focus on interactions among macroorganisms, such as competition (e.g. acidification influencing turfs-kelp interactions) and predation (e.g. warming leading to kelp grazing by range-expanding herbivorous fishes) [4,11], while interactions between macro- and microorganisms receive much less attention [12,13].

Despite their potential importance, little is known about the impacts of ocean climate change on interactions between macro- and microorganisms. While it is clear that microorganisms are critically important for the normal development and functioning of eukaryotic hosts [14], environmental stressors can disrupt the strong relationship between hosts and their associated microbiomes (defined here as an assemblage of microorganisms), leading to dysbiosis and host disease [15–17]. Hosts and their associated microbiomes form a coherent biological entity—‘holobiont’—that must be studied together to properly understand biological systems and how they will be impacted by climate change [14,18]. This is particularly crucial for habitat-forming organisms, because impacts on these holobionts can affect entire ecosystems [18].

On subtidal temperate rocky reefs, kelps (macroalgae of the order Laminariales) are the dominant habitat-forming species, providing food and shelter to many organisms, and playing a critical role in primary production and ecosystem functioning [19,20]. Key habitat-forming kelps are, however, declining in many places around the world due to impacts of multiple stressors, including climate change [8,18,19,21,22]. As a consequence, these systems typically shift from complex and productive forests to simpler, less productive habitats, with significant impacts on ecosystem services [18,23].

The kelp *Ecklonia radiata* (hereafter *Ecklonia*) forms extensive forests that dominate subtidal rocky reefs along 8000 km of temperate and sub-tropical Australian coastline [24–26], a region coined ‘the Great Southern Reef’ that provides ecosystem services with an estimated value of more than A$10 billion per year [27]. Critically, *Ecklonia* forests are declining in several areas across the continent due to direct and indirect effects of ocean warming, as well as poor water quality around urbanized shorelines [2,4,23]. These forests are predicted to decline further under future climatic conditions in response to warming and acidification [11,28,29].

Interactions between *Ecklonia* and its associated microbiome may contribute to, or even underpin, these declines. Recent surveys throughout the latitudinal distribution of *Ecklonia* identified putative disease phenotypes that were common and widespread. For example, kelp bleaching and associated tissue degradation were quantified in approximately 50% of the individuals in the populations sampled when water temperatures were warmest [30]. Bleaching in *Ecklonia* is related to changes in the associated microbiome and increases in abundances of putative pathogens, resulting in a lower photosynthetic capacity of the affected tissue [30]. Negative interactions between associated microbiomes and host health have also been observed in other, co-occurring Australian seaweeds (e.g. the red seaweed *Delisea pulchra* and the fucoid *Phyllospora comosa*) [31,32], where microbial diseases lead to striking changes in fecundity [33] or survival [32].

We experimentally tested the independent and interactive effects of ocean warming and acidification on the associated microbiome and condition of *Ecklonia*. We hypothesized that increases in water temperature and decreases in pH (simulating ocean acidification) would lead to changes in host-associated microbial communities and to diminished condition of the host (i.e. lower photosynthetic efficiency or disease symptoms similar to those observed in the field [30]). We found that tissue of some kelp blistered and subsequently bleached and degraded during the experiment as a result of warming and/or acidification. We then characterized the structure of the microbiome associated with blistered and healthy kelp to examine effects of kelp condition, warming and acidification on microbiomes.

## 2. Material and methods

### (a) Mesocosm experiment

*Ecklonia* individuals (sub-adults, mean biomass 162 ± s.e. 13 g; maximum length approximately 30–60 cm) were haphazardly collected from Charlesworth Bay (30.2677° S, 153.1435° E), Coffs Harbour, Australia, by detaching the holdfasts from the substratum. All collected individuals appeared healthy with no obvious signs of disease or stress. *Ecklonia* individuals were exposed to warming and acidification in twelve flow-through, fibreglass mesocosms (1100 l, 1.35 m diameter × 0.90 m high) located outdoors and under a shade cloth at the National Marine Science Centre (30.3022° S, 153.1189° E), Coffs Harbour, Australia (see [11]). The experiment included two orthogonal factors: *warming* (current [approximately 21°C] versus future [approximately 23.5°C]) and *acidification* (current [pH_NIST_ = approximately 8.17, pCO_2_ = approximately 400 µatm] versus future [pH_T_ = approximately 7.97, pCO_2_ = approximately 685 µatm]; details in electronic supplementary material, table S1), with each combination of treatments randomly assigned to 3 mesocosms. Ambient conditions represented the approximate average ocean conditions when the experiment was undertaken (July–early August 2014, http://www.metoc.gov.au/) and the future conditions corresponded to RCP 8.5 model predictions for 2081–2100 [34].

Each of the 12 mesocosms housed six kelp. The holdfast of each kelp was attached to mesh at the base of a mesocosm with cable-ties fed through silicone tubing to avoid stipe damage [35,36]. Each mesocosm received filtered seawater (50 µm) at 3 l min^−1^ that was pumped from the adjacent ocean, with the inlet located at the site of kelp collection. This flow-through rate ensured that the entire volume of each mesocosm was replaced multiple times a day. Thus, microorganisms in the water would have been continuously refreshed throughout the experiment as in natural conditions. Water temperature was controlled using heater/chiller units (Aquahort Ltd) and pH was manipulated by bubbling in ambient air or CO_2_-enriched air via a gas mixer (PEGAS 4000MF). The mesocosms experienced natural daily fluctuations in temperature and pH, which varied consistently across treatments, maintaining treatment differences (electronic supplementary material, table S1). Water temperature, conductivity and pH were measured daily in each mesocosm using a Hach HQ40d multi probe calibrated with NIST buffers. Total alkalinity (*A*_T_) for the system was also measured daily using Hg fixed samples and a potentiometric titration (888 Titrando, Metrohm, USA). The partial pressure of carbon dioxide (pCO_2_), the saturation states of calcite (*Ω*_calc._) and aragonite (*Ω*_arag._) and the concentrations of carbonate $\left(CO_{3}^{2-}\right)$ and bicarbonate $\left(\mathrm{HC}O_{3}^{-}\right)$ were calculated from the *A*_T_, pH_NIST_ and temperature measurements with constants from Mehrbach *et al.* [37] as adjusted by Dickson & Millero [38], and average local salinity during the time of the experiment (35.6 ppt, http://www.metoc.gov.au/).

### (b) Sampling microbiomes and kelp condition

Microbial communities on the surface of kelp across all treatments were sampled via swabbing each of 2 haphazardly chosen kelp individuals per mesocosm (*n* = 6 per treatment) at 8, 16 and 31 days after the experiment commenced (*N* = 72), ensuring independent samples of visually healthy tissue through time. An area of 20 cm^2^ on the middle section of a secondary lamina (approx. 15 cm from the meristem) was briefly rinsed with filtered (0.22 µm PES syringe filter, Millipore) seawater and swabbed using an autoclaved sterile cotton tip for 30 s [30,39]. The swabs were immediately transferred into sterile, DNA-free cryogenic tubes (SSIbio, Scientific Specialties Inc.) and stored at −80°C until DNA extraction. DNA of some samples did not amplify after PCR, resulting in 4–6 replicates per treatment (i.e. 1–2 replicates per mesocosm), totalling 66 samples.

**Figure 1.**
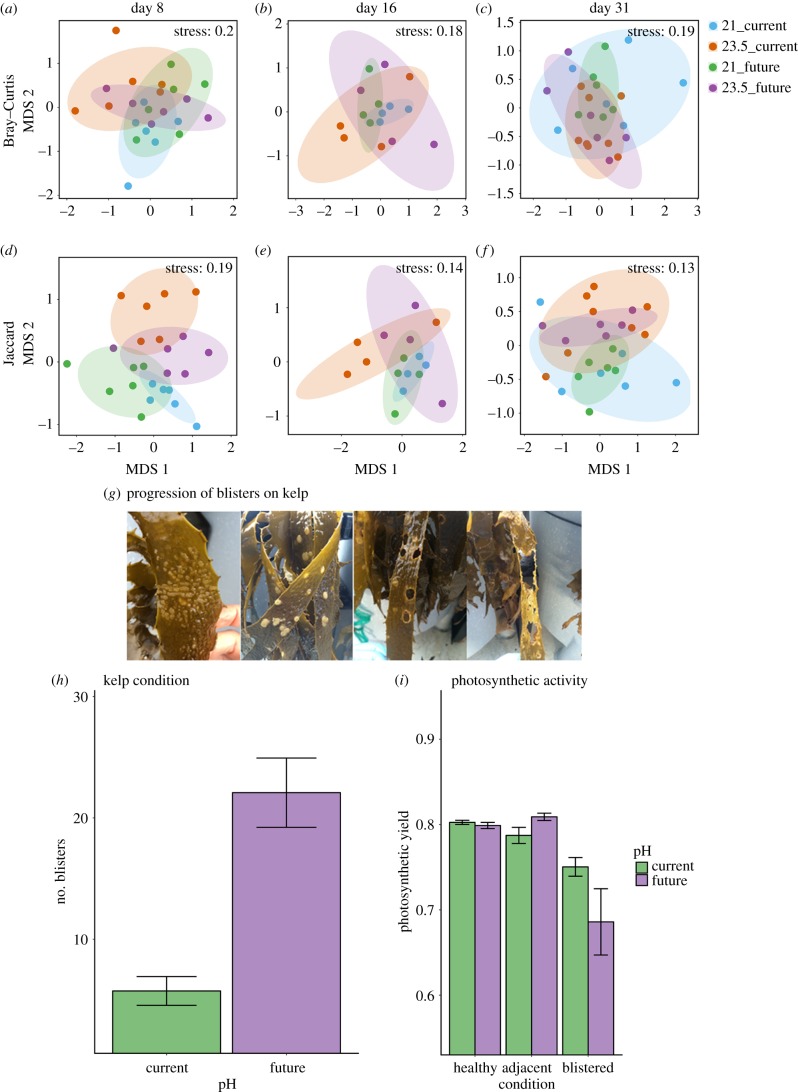
Effects of warming and acidification on kelp microbiomes and development, prevalence and functional effects of kelp blistering in response to acidification. nMDS based on the Bray–Curtis (*a–c*) or Jaccard (*d–f*) measures on square-root transformed relative abundances of microorganisms on healthy kelp across all pH (*a*,*b*; 8.17 ‘current’ versus 7.97 ‘future’) and temperature (*c*,*d*; 21 versus 23.5°C) treatments at three time-points (days 8, 16 and 31) during the experiment. Larger, shaded ellipses show the grouping of each treatment (blue: 21°C current, orange: 23.5°C current, green: 21°C future, purple: 23.5°C future). (*g*) Photographs of kelp laminae showing the progression of blistering of the tissue throughout the experiment. (*h*,*i*) Mean (±s.e.) number of blisters on individual kelp laminae (*h*; *n* = 54) and maximum photosynthetic yield of healthy, adjacent to blistered or blistered tissue (*i*; *n* = 6–8) under ‘current’ (green bars) and ‘future’ (purple bars) pH treatments.

On the second week of the experiment, we observed blistering of the tissue on sections of the secondary lamina of several kelp individuals (figure 1*g*). We quantified and compared the prevalence of this putative disease across different treatments by counting the number of blisters on three haphazardly chosen secondary laminae of each of three kelps in each mesocosm (i.e. *n* = 9 kelp per treatment). To test for effects on kelp photosynthetic efficiency, the maximum photosynthetic quantum yield (i.e. the maximal light utilization efficiency in the dark; *Fv*/*Fm*) of healthy, blistered and immediately adjacent to blistered tissues, from secondary laminae from each of 2–4 kelp per condition and temperature × acidification treatment combination was quantified using a pulse amplitude modulated fluorometer (PAM; Walz, Germany) [30].

To compare microbial communities on healthy and blistered kelps, one independent healthy or blistered section of secondary laminae, each from different kelp individuals, was swabbed on day 16 as described above. Swabs were taken from four plants per condition, haphazardly chosen across mesocosms in each treatment. By the last sampling time (day 31), the sections of blistered tissue had grown, often coalesced, bleached as observed in previous field sampling [30], and subsequently decomposed and sloughed off, so we were unable to sample blistered kelp at this time.

### (c) DNA extraction and sequencing

Swabs were transferred to the University of New South Wales (UNSW) in sterile cryo-tubes within liquid nitrogen. Microbial DNA was extracted from each swab using a 96-well PowerSoil DNA Isolation Kit (MO Bio Inc., Carlsbad, CA, USA) following the manufacturer's protocol. DNA extracts were qualified and quantified by agarose gel electrophoresis and spectrophotometry (NanoDrop 1000) and stored at −20°C. Samples were processed and sequenced using standard aseptic procedures and in a random order to avoid contamination and introducing any bias due to order of processing.

The extracted microbial DNA samples were amplified with PCR using the 16S rDNA primers 27F (5′-AGAGTTTGATCMTGGCTCAG-3′) and 519R (5′-GWATTACCCGCGGCKGCTG-3′), containing V1 to V3 regions of the bacterial and archaeal 16S rRNA gene. PCR controls (no loaded sample) did not amplify DNA, suggesting no contamination during processing and amplification. Amplicons were purified (Zymo DNA-5 Clean Concentrator) before sequencing via the Illumina MiSeq 2000 platform at the Ramaciotti Centre for Genomics (UNSW).

### (d) 16S rRNA gene processing and quality filtering

Raw sequencing data were quality filtered, standardized, classified and then clustered into operational taxonomic units (OTUs) using Mothur [40]. Forward and reverse reads (301 bp) were combined into contigs. Sequences that contained unidentified bases or had greater than 8 homopolymers were filtered out. Remaining sequences were aligned referring to the Silva 16S rRNA gene database [41]. Sequences that did not align were excluded. Aligned sequences were pre-clustered (diffs = 2) and checked for chimeras using UCHIME [42]. Singleton and doubleton sequence reads were removed from the dataset to reduce error [43]. Sequences were then taxonomically classified according to the Silva database with 60% cut-off confidence and clustered into OTUs at a minimum of 97% sequencing identity, resulting in a total of 8401 OTUs. Sequence counts were rarefied to 46 473 reads (total number of high-quality sequences obtained) per sample to account for differences in sequencing depth. Rarefaction curves of the processed sequences were asymptotic, suggesting good coverage of the microbial diversity present (electronic supplementary material, figure S1). To focus analyses on abundant OTUs and reduce the effect of potentially spurious OTUs, those that contributed less than 0.01% of relative abundance were removed from the dataset, resulting in 1887 OTUs for further analyses.

### (e) Statistical analysis

The OTU matrix was analysed using permutational multivariate analysis of variance (PERMANOVA) [44] in PRIMER v. 6 (PRIMER-E, UK) which compared bacterial and archaeal communities (*i*) on healthy kelp across treatments for all time points, (*ii*) between condition (healthy versus blistered) and treatments at day 16. The first analysis had temperature (21 versus 23.5°C) and acidification (‘current’ versus ‘future’) as fixed factors and time as a random, orthogonal factor (days 8, 16, 31). The second analysis had kelp condition (healthy versus blistered), temperature and acidification as fixed factors. Random effects of mesocosms were not considered because we only had mostly 1 (sometimes 2) replicates per mesocosm for each treatment combination due to low DNA yield of some samples, so the mesocosms were essentially the replicates. Similarity matrices were calculated based on Bray–Curtis distances on square-root transformed abundance data (‘community structure’), and on Jaccard distances (presence/absence; ‘community composition’). Analyses used 9999 permutations of residuals under a reduced model [45]. Permutational multivariate dispersion (PERMDISP) analysis was used to test for homogeneity of multivariate dispersion within groups [46]. Non-metric multidimensional scaling (nMDS) ordinations allowed visualization of microbial community structure under different treatments.

We compared abundances of each OTU across the different treatments and kelp conditions using multivariate generalized linear models (GLM) assuming a negative binomial distribution in the R statistical package mvabund [47]. Residual plots were checked to ensure good model fit [47]. Dozens to hundreds of OTUs were found to significantly differ among the treatments (see Results; electronic supplementary material, tables S2 and S3). We focused on those OTUs where the effect of warming, acidification, kelp condition and/or their combination was strongest, defined here as those for which the absolute effect size was greater than twice the standard deviation (s.d.).

Univariate generalized linear mixed models (GLMM) were used to compare the number of blisters on kelp among treatments, and the photosynthetic yield of kelp among conditions (healthy, blistered and adjacent to blisters) and treatments at day 16. The numbers of blisters were modelled assuming a Poisson distribution (count data) and photosynthetic yields were modelled assuming a gamma distribution (positive numbers) using the R statistical package lme4 [48]. Significance was tested using Wald chi-square (*χ*^2^) tests. Mesocosm and kelp individuals were added as random effects in the models to account for potential within-unit correlations. Residual plots were checked to ensure good model fit and the absence of overdispersion [49].

## 3. Results

### (a) Effect of ocean warming and acidification on healthy kelp microbiomes

On healthy kelp, microbial community structure (identity and relative abundances) and composition (identity only) generally differed between temperature treatments at the start (day 8; figure 1*a*,*d*) and end (day 31; figure 1*c*,*f*) of the experiment, although no significant differences were found at day 16 (figure 1*b*,*e*; electronic supplementary material, table S4). Acidification significantly affected microbial community composition at the start of the experiment (day 8; figure 1*d*; electronic supplementary material, table S4).

GLM analyses identified 46 OTUs (approx. 2% of a total of 1887 OTUs) whose relative abundances differed significantly among temperature and/or acidification treatments (electronic supplementary material, table S2). Of those, we found a strong (greater than 2 s.d.; Material and methods) main effect of temperature on 4 OTUs that belong to the classes Bacteroidetes and Alphaproteobacteria, with abundances generally lower on kelp at 23.5°C (electronic supplementary material, figure S2a). Three OTUs were strongly affected by acidification, but the direction of the effect varied, e.g. an OTU belonging to the genus *Aquimarina* was more abundant in the acidification treatments, while the opposite pattern was found for an OTU belonging to the genus *Glaciecola* (electronic supplementary material, figure S2b). We found a strong interactive effect of warming and acidification on 12 OTUs; generally, these OTUs were found in higher abundances on kelp under the higher temperature (e.g. those belonging to the families Rhodobacteraceae and Polaribacter) and/or future pH treatments (e.g. those belonging to the families Flavobacteriaceae and Flammeovirgaceae; electronic supplementary material, figure S2c and table S2).

### (b) Kelp condition and associated microbial communities

During the second week of the experiment, we observed kelps with clusters of blisters on the tissue of their primary and secondary laminae, which subsequently coalesced and led to tissue bleaching and degradation (figure 1*g*) as previously observed in the field. On day 16, the number of blisters (22 ± s.e. 2) was three to four times higher on kelp subjected to ocean acidification compared to ambient conditions (6 ± s.e. 1; figure 1*h*). No significant differences were found in the number of blisters at different temperatures (electronic supplementary material, table S5a). Blistered tissue had a lower photosynthetic yield than healthy tissue adjacent to the blisters or healthy tissue from kelp without blisters, and this localized effect was strongest for kelp under the acidification treatments (figure 1*i*; electronic supplementary material, table S5b). Warming had no influence on kelp photosynthetic yield (electronic supplementary material, table S5b).

**Figure 2.**
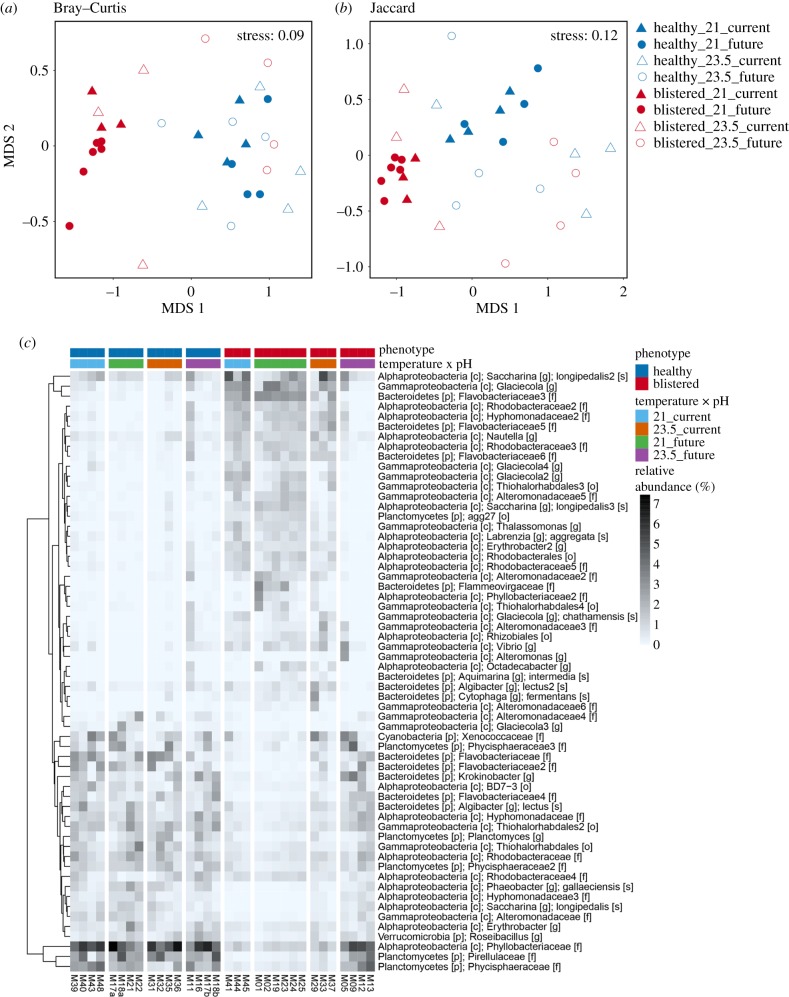
Differences in kelp microbiomes on healthy and blistered kelp under warming and acidification. nMDS based on the Bray–Curtis (*a*) or Jaccard (*b*) measure of square-root transformed relative abundances of OTUs on ‘healthy’ (blue symbols) versus ‘blistered’ (red symbols) kelp under the temperature (21 versus 23.5°C, filled or empty symbols, respectively) and pH (‘current’ versus ‘future’, triangles or circles, respectively) treatments at day 16. (*c*) Heatmap of relative abundances (square-root transformed) of all OTUs that differed significantly between ‘healthy’ versus ‘blistered’ kelp samples at day 16 in at least one temperature (21 versus 23.5°C) × pH (‘current’ versus ‘future’) treatment combination.

Microbial community structure on blistered kelp differed significantly from that on healthy kelp across all treatments, except for those at future temperature and acidification, which showed a similar structure to healthy kelp (electronic supplementary material, table S6). This pattern was mainly due to differences in relative abundances of OTUs (figure 2*a*), as differences in composition were only found for communities at 21°C under acidification (figure 2*b*; electronic supplementary material, table S6). Temperature only affected microbial community structure and composition on blistered kelp subjected to acidification. There was also an effect of acidification on microbial community structure, but only on blistered kelp at 23.5°C (electronic supplementary material, table S6).

Univariate analyses identified *ca* 450 OTUs (approx. 24%) that differed in abundance between healthy and blistered samples in at least one temperature × acidification treatment combination (electronic supplementary material, table S3), with a strong effect size for 61 of these OTUs (figure 2*c*). However, for most OTUs on kelp under the future temperature and acidification combination, abundances on blistered samples did not differ from those on healthy samples (figure 2*c*). Some bacterial taxa were consistently dominant on blistered (e.g. OTUs belonging to the genus *Alteromonas* or family Flavobacteriaceae) or healthy (OTUs assigned the genera *Roseibacillus* or *Planctomyces*) kelp, independently of the pH and temperature treatments (electronic supplementary material, figure S3a and table S3). Several taxa were also influenced by pH, but the effect was variable: taxa were either found in higher abundances on healthy kelp under both current and future pH (family Pirellulaceae) or just future pH (genus *Erythrobacter*), or on blistered kelp under current pH (OTUs assigned to the species *Glaciecola chathamensis*; electronic supplementary material, figure S3b). For most microbial taxa, however, differences in relative abundance between kelp conditions were influenced by temperature (electronic supplementary material, figure S3c and table S3). In most cases, abundances were significantly lower on blistered kelp, particularly at 21°C, although some taxa (e.g. OTUs belonging to the genus *Thalassomonas* or family Alteromonadaceae) were present in higher abundances on blistered kelp at 21°C than in all other treatments (electronic supplementary material, figure S3c).

## 4. Discussion

Microbiomes of habitat-formers may be impacted by ocean climate change with implications for the health, persistence and resilience of entire marine ecosystems. We showed that ocean warming and acidification can rapidly affect kelp-associated microbiomes, kelp condition and performance. Kelp individuals developed blisters on the laminae and this putative disease was more prevalent on individuals under acidification treatments. Blistered tissues had lower photosynthetic efficiency and subsequently bleached, similar to phenotypes previously observed in the field [36], and degraded. Given that changes in the microbiomes were rapid and preceded the observed changes in the tissue by several days, this suggests that predicted ocean warming and, in particular, acidification may have strong negative impacts on populations of *Ecklonia* via disruptions of its associated microbiome. Given the ecological role that this species plays along the temperate Australian coastline [27], such impacts may affect entire coastal ecosystems.

The prevalence of blisters on kelp was strongly influenced by acidification. This may be due to direct, physiological effects of this stressor on the kelp, which could have led, in turn, to changes in the microbiome and disease, although some blistering was also observed in other treatments albeit in significantly lower numbers. Alternatively, changes in the microbiome may have been a direct response to acidification at the beginning of the experiment, and may have subsequently led to blistering of the kelp. Changes in kelp-associated microbiomes were observed before changes in the condition of the kelp, suggesting an indirect effect of acidification on kelp condition, that is, via changes in the microbiome.

Of particular interest was, however, the finding that microbial community structure associated with blistered tissue differed markedly from that associated with healthy tissue in most treatments except for the combination of future temperature and acidification (i.e. the future scenario), where microbiomes resembled those on healthy kelp. These results may be caused by ocean warming and acidification having independent, yet antagonistic effects on microbial communities. As more studies simultaneously test for the impacts of ocean warming and acidification using factorial designs, it is becoming increasingly apparent that warming and acidification have independent effects on biological response variables, rather than interactive effects (e.g. [11]). When the direction of the effects of ocean warming and acidification oppose each other, they tend to moderate, rather than exacerbate, the impacts of ocean climate change [50]. While these types of opposing effects have been demonstrated for univariate biological response variables (e.g. coralline growth/health [50]), our data suggest that this could also be the case for entire microbial communities. Alternatively, it is possible that the microorganisms initially responsible for affecting the condition of the host were unable to withstand the combination of warming and acidification. This model is interesting as it implies that some of the currently known marine microbial pathogens may be less infective (or not infective at all) under future, more stressful environmental conditions [51]. However, the exposure to such conditions will be gradual, rather than abrupt as in this short-term experiment, allowing for evolutionary processes to take place [15]. Indeed, microbial communities can have the capacity to adapt rapidly to changing environmental conditions [52]. It is therefore possible that both host and microbiome may gradually adapt to the future ocean warming and acidification conditions modelled in this study, making predictions based on short-term studies such as this one challenging. Small-scale, short-term experimental manipulations are a powerful tool to determine causality, but it is hard to generalize observed effects in such experiments, particularly where other trophic levels and/or large-scale processes (e.g. ocean currents/eddies) are ignored. Combining short-term experiments with longer-term field sampling, or using space-for-time substitutions at appropriate locations [4], can help to reduce uncertainties and improve forecasting.

Environmental changes have been linked to microbial diseases in other key habitat-formers including corals [15,53], and our understanding of negative host–microbiome interactions on seaweeds is increasing [31,54]. Microbial diseases have recently been characterized in stressed populations of habitat-forming seaweeds in SE Australia, including the red alga *Delisea pulchra* (bacterial disease) and the canopy-forming fucoid *Phyllospora comosa* (endophytic fungal disease), in response to warming and around urbanized areas [31–33,55]. Such diseases can affect survival, photosynthetic efficiency, fecundity and influence greater consumption and host-use by herbivores [33]. In previous studies, we have identified putative pathogens and found a strong relationship between the structure and composition of microbiomes and host performance for *Ecklonia* throughout its latitudinal distribution in Australia [30], combined with an increase in kelp bleaching with temperature [30,56]. Thus, ocean warming may lead to increased frequency and severity of bleaching in *Ecklonia* via direct physiological impacts combined with a disruption of the microbiome. Apart from observing changes in microbiomes in response to warming in the present experiment, we did not detect any measurable effects of temperature on the prevalence of kelp blistering (numbers of blisters). This could be partly a function of quantifying the number of blisters rather the overall area of tissue impacted because, as the blisters grew in size, they coalesced with other blisters to form larger affected bleached areas similar to those observed in the field when water temperature is warmest [30]. Thus, quantification of the total area affected may have also potentially revealed an effect of temperature. Alternatively, this could be due to kelp already experiencing similar temperatures at the end of summer or during localized heatwaves. Kelp may be more resilient to warming than acidification, although recent studies show that exposure to warm temperatures similar to the one used here can affect kelp condition and survival leading to significant kelp declines [2,4,30]. Additionally, different stages in the life history of kelp are likely to respond to environmental changes differently, and thus responses by adults may not reflect those by early life stages (e.g. recruits), which can be less tolerant.

The putative disease—blistering, bleaching, tissue degradation—observed here could have resulted from a mesocosm effect (i.e. moving kelp from the natural environment into mesocosms), although numbers varied among treatments. We were not able to sample field populations at the time of the experiment to assess blistering or bleaching levels; however, we have observed kelp bleaching and tissue degradation in many *Ecklonia* populations along the continent [30], not related to photobleaching or tissue loss following reproduction, but which resembles the bleaching and degradation that followed the blistering in this experiment. Similar diseases have been observed in other seaweeds in the field, for example gall/blister development and discoloration on fronds of the fucoid *Durvillaea antarctica* in Chile in response to parasite infections [57]. This and the higher prevalence of blistering and subsequent bleaching of kelp in treatments simulating future ocean conditions suggest that the putative disease observed in our mesocosm experiment is likely to occur in natural populations. Alternatively, kelp may be primarily influenced by acidification rather than temperature, and may only become prevalent in the field once the ocean becomes more acidic. As with all mesocosm experiments, interpretation of results may be affected by the nature of the mesocosm, relative to the natural environment. Here, some effects could have been due to an increased probability of colonization of pathogens from the water (which were not monitored) onto the kelp surfaces, such as if warming and/or acidification would have led to an increase in water pathogens. However, any such confounding issues are unlikely for several reasons. First, previous studies have shown very distinct communities in the water from those associated to any live surface, including *Ecklonia* [57]. It is unlikely therefore that changes in the microorganisms in the water, if any, would have resulted in changes in the surface-associated communities. Second, the entire volume of each mesocosm was replaced multiple times a day with water collected from the kelp-collection site. Thus, microorganisms in the water would have been continuously refreshed, reflecting natural changes in the site where the kelp was collected. In addition, differences between treatments were due to differences in relative abundances of taxa already present on the kelp surfaces, rather than differences in presence or absence of taxa.

Differences among treatments were driven by abundances of approximately 20% of the OTUs, of which even fewer showed large effect sizes (less than 5%). The presence of a small ‘core’ set of taxa associated with differences in host properties is consistent with other studies on marine microbial diseases [30] and on seaweed-associated microbial communities in general [58,59]. Several taxa identified belonged to genera or families of marine pathogens associated with bleached *Ecklonia* and/or *Delisea* [30,60], as well as diseased sponges and corals [12,61] (e.g. *Vibrio*, *Alteromonas*, *Aquimarina*, Flavobacteriaceae and Rhodobacteriaceae), suggesting that multiple taxa may cause changes in host condition. Some of these strains have been used in inoculation experiments with *Delisea* which showed that multiple opportunistic pathogens can cause bleaching disease [60]. Similar experiments are necessary to establish a causal link between the observed changes in abundances of these and other taxa, and functional changes and the development of blistering in *Ecklonia*. Although our focus has been on surface-associated microbiomes, it is possible that endophytic microbes may also be responsible for the blistering/bleaching or other diseases [32,57], but little is known in this respect for *Ecklonia*.

Contamination can be an issue in microbial studies. We controlled for this in part by PCR (negative) controls and the use of sterile equipment and aseptic procedures, which in amplicon sequencing studies reduce the likelihood of contamination, and are standard and accepted practice in mensurative or manipulative studies [30,58,62]. In our experiment samples were collected, extracted, amplified and sequenced randomly; thus, any potential contamination in the sterile commercial kits or throughout this process would have affected all treatments in an unbiased way. The specifics of our results also argue against confounding by contamination. For example, for such contamination to influence the reversion of treatments under future climatic conditions to current ones, there would have to have been contamination from marine bacteria at a sufficient level in pre-sterilized kits in all samples of the future conditions treatment, that matched the specific community composition of the communities in the treatments subject to current climatic conditions. Therefore, it is very unlikely that observed differences of communities/taxa among treatments were due to contamination.

Host-associated microbial communities can have critical roles in the normal development, functioning and defence of marine organisms [15,16,63]. There is increasing evidence that disruptions of ecological interactions between hosts and their microbiomes can negatively impact host health and performance, reinforcing the idea that these biological associations (host and microorganisms) should be studied holistically, as ‘holobionts’ [14–16]. This is particularly important for habitat-forming holobionts that form the biogenic structure of ecosystems (e.g. trees, kelp, corals) because impacts on the interaction between these hosts and their microbiomes can affect entire ecosystems. Such habitat-forming species are in significant decline [4,22], and this is likely to be exacerbated due to changes in ocean climates [2,28]. Although we are beginning to gain a mechanistic understanding of the indirect effects of climate change via changes in ‘macrobial’ species' interactions [11,64], we still know very little about how host–microbiome interactions affects resilience to ocean warming and acidification. Such understanding is, however, crucial in order to provide early warning signals and conserve and manage key marine ecosystems that underpin temperate reef biodiversity and functioning.

## Supplementary Material

Fig.S2.Microbial taxa affected significantly by warming and acidification.

## Supplementary Material

Fig.S3.Microbial taxa affected significantly by kelp condition, warming and acidification.

## Supplementary Material

Mesocosm characteristics, rarefaction curves and multivariate analyses

## Supplementary Material

Microbial taxa affected significantly by warming and acidification and/or condition.

## Data accessibility

Raw sequences are available through the Sequence Read Archive, NCBI (submission ID: SUB3754286) and in the Dryad Digital Repository: https://doi.org/10.5061/dryad.p8br3r4 [65].
