# Supplementary figures and images for: Future climate change is predicted to affect the microbiome and condition of habitat-forming kelp
Source: Proc Biol Sci. 2019 Feb 6;286(1896):20181887. doi: 10.1098/rspb.2018.1887 (PMC6408609; doi:10.1098/rspb.2018.1887)

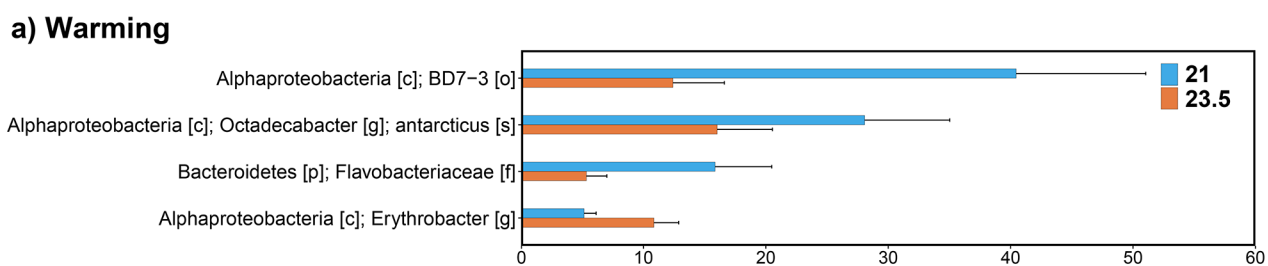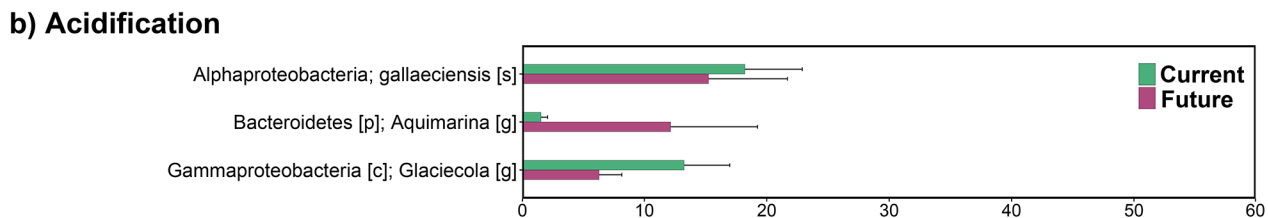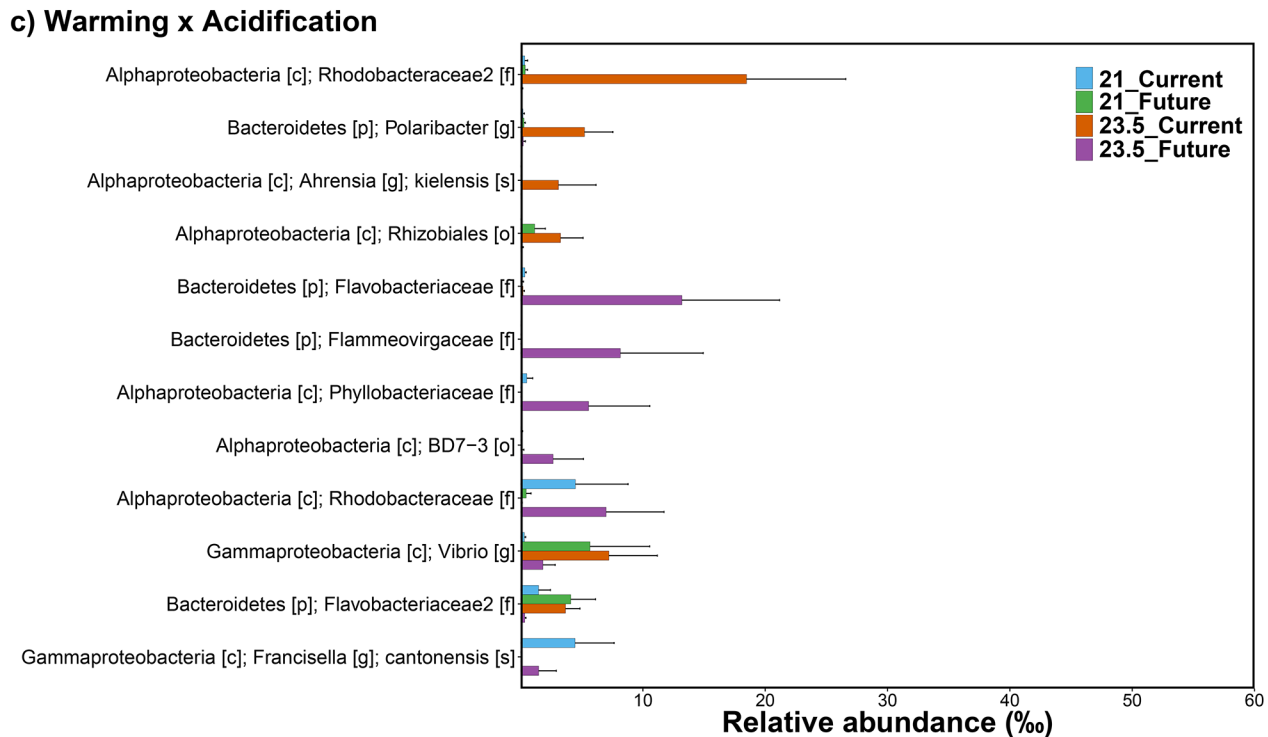

Supplement: Fig.S2.Microbial taxa affected significantly by warming and acidification. [file rspb20181887supp1.pdf]

## a) Condition

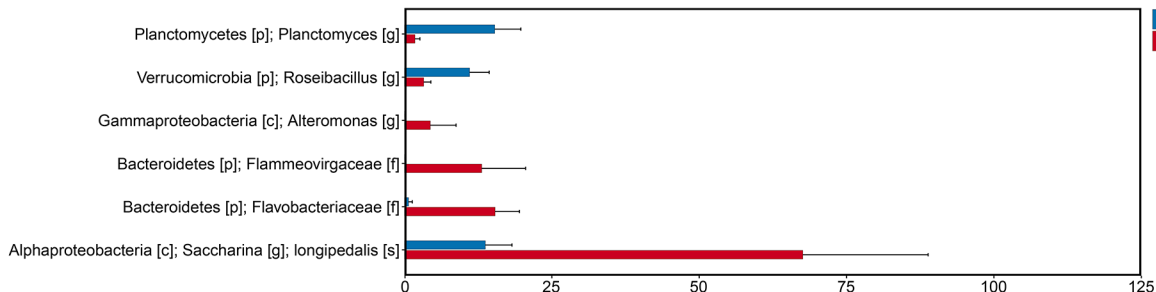

## b) Condition x Acidification

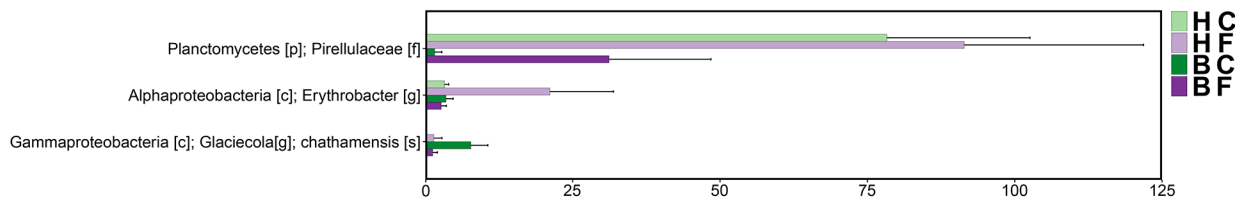

## c) Condition x Warming

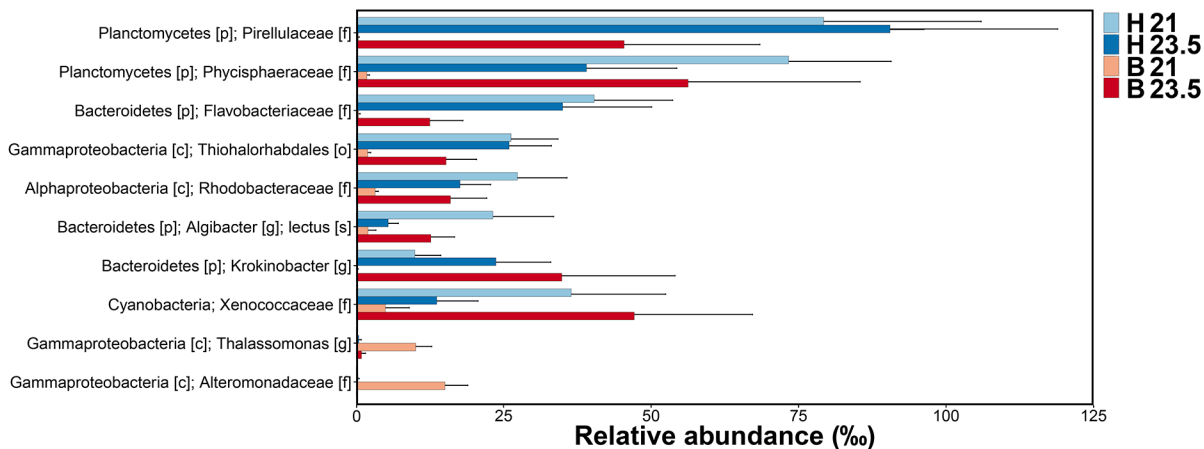

Supplement: Fig.S3.Microbial taxa affected significantly by kelp condition, warming and acidification. [file rspb20181887supp2.pdf]
