# Supplementary material for: Future climate change is predicted to affect the microbiome and condition of habitat-forming kelp
Source: Proc Biol Sci. 2019 Feb 6;286(1896):20181887. doi: 10.1098/rspb.2018.1887 (PMC6408609; doi:10.1098/rspb.2018.1887)
Supplement: Mesocosm characteristics, rarefaction curves and multivariate analyses [file rspb20181887supp3.docx]

**Electronic Supplementary Material – Table S1, Fig. S1, Tables S4, S5, S6, Figs. S3, S4**

Title: Future climate change is predicted to affect the microbiome and condition of habitat-forming kelp

Authors: Zhiguang Qiu, Melinda A. Coleman, Euan Provost, Alexandra H. Campbell, Brendan P. Kelaher, Steven J. Dalton, Torsten Thomas, Peter D. Steinberg, Ezequiel M. Marzinelli

Journal: Proc. R. Soc. B

DOI: 10.1098/rspb.2018.1887

**Table S1:** The average (± SD) measured and calculated seawater conditions in the mesocosms. Data include water temperature (T), pH_NIST_, partial pressure of carbon dioxide (*p*CO_2_), the saturation states of calcite (Ω_calc._) and aragonite (Ω_arag._) and the concentrations of carbonate (CO_3_ ^2–^) and bicarbonate (HCO_3_^–^).

| **Treatment** | **T**  (°C) | ***p*H_NIST_** | ***p*CO_2_**  (µatm) | **Ω_calc._** | **Ω_arag._** | **CO_3_ ^2–^** (µmol.kgSW^-1^) | **HCO_3_^–^** (µmol.kgSW^-1^) |
| --- | --- | --- | --- | --- | --- | --- | --- |
| 21°C & Ambient CO_2_ | 20.85 ± 0.01 | 8.17 ± 0.01 | 391.08 ± 12.30 | 4.53 ± 0.09 | 2.96 ± 0.06 | 190.57 ± 3.93 | 1806.48 ± 9.75 |
| 23.5°C & Ambient CO_2_ | 23.54 ± 0.14 | 8.16 ± <0.01 | 411.06 ± 0.98 | 4.75 ± 0.02 | 3.12 ± 0.01 | 198.92 ± 0.83 | 1786.79 ± 2.01 |
| 21°C & Future CO_2_ | 21.22 ± 0.18 | 7.96 ± 0.02 | 704.26 ± 34.95 | 3.03 ± 0.10 | 1.98 ± 0.06 | 127.17 ± 4.09 | 1963.85 ± 10.18 |
| 23.5°C & Future CO_2_ | 23.55 ± 0.42 | 7.99 ± <0.01 | 662.54 ± 6.13 | 3.42 ± 0.05 | 2.25 ± 0.04 | 143.29 ± 2.03 | 1924.67 ± 4.91 |

Calculations were made using constants from Mehrbach *et al.* (1973), as adjusted by Dickson and Millero (1987), and the average total alkalinity (2278.0 ± 39.1 µmol.kgSW^-1^) and salinity (35.6 ppt) for the system.


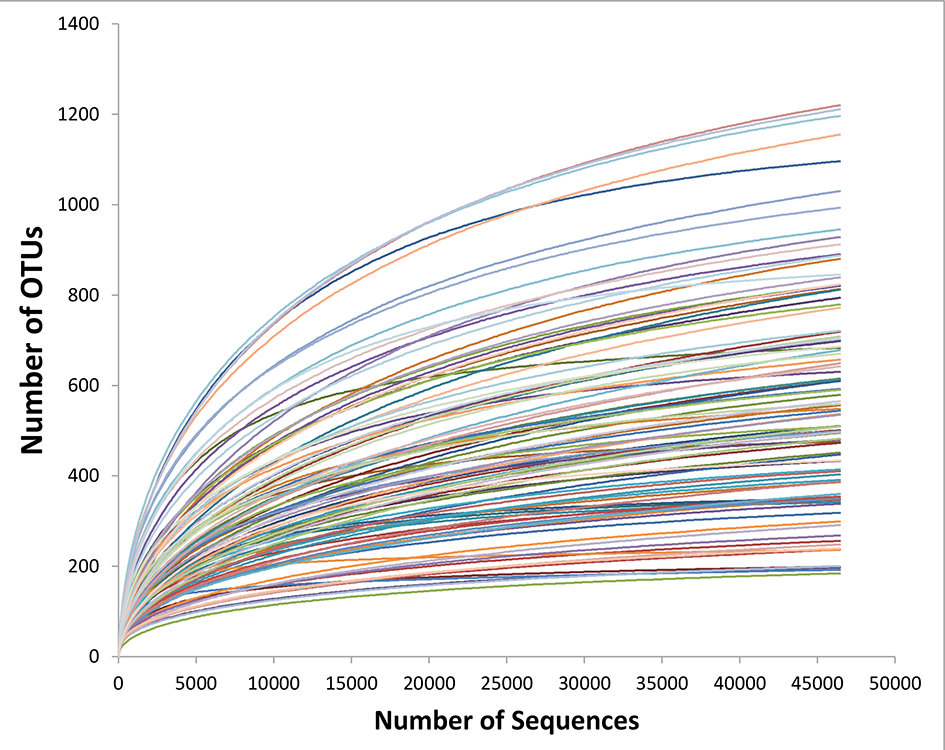


**Fig. S1.** Rarefaction curves for the 46,473 high-quality sequences of kelp microbiomes obtained via 16S rRNA gene tag sequencing.

**Table S4.** PERMANOVA analyses based on Bray-Curtis (a) and Jaccard (b) measures of square-root transformed relative abundances of microorganisms on healthy kelp under temperature (Te; fixed, 21^o^C vs 23.5^o^C) and pH (fixed, 8.17 = current ‘Cu’ vs 7.97 = future ‘Fu’) treatments at three sampling times (Ti; random, 8, 16 and 31 days).

|  |  | (a) Bray Curtis | | | (b) Jaccard | | |
| --- | --- | --- | --- | --- | --- | --- | --- |
| Source | df | MS | pseudo-*F* | *p* | MS | pseudo-*F* | *p* |
| Te | 1 | 3297 | 1.47 | 0.317 | 4915 | 1.25 | 0.344 |
| pH | 1 | 2750 | 1.47 | 0.303 | 3882 | 1.16 | 0.339 |
| Ti | 2 | 14576 | 8.87 | **<0.001** | 10878 | 3.66 | **<0.001** |
| Te x pH | 1 | 2560 | 1.38 | 0.331 | 4048 | 1.18 | 0.385 |
| Te x Ti | 2 | 2252 | 1.37 | **0.011** | 3963 | 1.33 | **<0.001** |
| pH x Ti | 2 | 1874 | 1.14 | 0.155 | 3368 | 1.13 | 0.057 |
| Te x pH x Ti | 2 | 1862 | 1.13 | 0.168 | 3437 | 1.16 | **0.037** |
| Residual | 54 | 1644 |  |  | 2972 |  |  |
| Pairwise tests: | |  |  |  |  |  |  |
| Te x Ti | | d.8 & 31: 21≠23.5^o^C  d.16: 21=23.5^o^C | | |  | | |
| Te x pH x Ti, for Te | |  | | | d.8, Cu & Fu: 21≠23.5^o^C  d.16, Cu & Fu: 21=23.5^o^C  d.31, Cu: 21=23.5^o^C, Fu: 21≠23.5^o^C | | |
| Te x pH x Ti, for pH | |  | | | d.8, 21 & 23.5^o^C: Cu≠Fu  d.16 & 31, 21 & 23.5^o^C: Cu=Fu | | |

**Table S5.** (a) Analysis of number of blisters on kelp for each temperature (Te; 21^o^C vs 23.5^o^C) and pH (8.17 = current ‘Cu’ vs 7.97 = future ‘Fu’) treatments at day 16. (b) Analysis of maximum photosynthetic yield on healthy (H) vs adjacent to blistered (A) vs blistered (B) kelp condition (Co) for each temperature and pH treatments at day 16. Both analyses included mesocosm and kelp individual as random effects.

| (a) Number of blisters | | | | (b) Photosynthetic yield | | | |
| --- | --- | --- | --- | --- | --- | --- | --- |
| Source | df | *X^2^* | *p* | Source | df | *X^2^* | *p* |
| Te | 1 | 3.70 | 0.074 | Te | 1 | 0.73 | 0.392 |
| pH | 1 | 6.44 | **0.011** | pH | 1 | 0.81 | 0.368 |
| Te x pH | 1 | 0.82 | 0.364 | Co | 2 | 30.79 | **<0.001** |
| Residual | 102 |  |  | Te x pH | 1 | 0.13 | 0.719 |
|  |  |  |  | Te x Co | 2 | 3.03 | 0.221 |
|  |  |  |  | pH x Co | 2 | 9.10 | **0.011** |
|  |  |  |  | Te x pH x Co | 2 | 0.38 | 0.823 |
|  |  |  |  | Residual | 29 |  |  |
|  |  |  |  | Pairwise tests:  Cu & Fu: H = A > B  H & A: Cu = Fu; B: Cu > Fu | | | |

**Table S6.** PERMANOVA analyses based on (a) Bray-Curtis and (b) Jaccard measures of square-root transformed relative abundances of microorganisms on healthy (H) vs blistered (B) kelp condition (Co; fixed) for each temperature (Te; fixed, 21^o^C vs 23.5^o^C) and pH (fixed, 8.17 = current ‘Cu’ vs 7.97 = future ‘Fu’) treatments at day 16.

|  |  | (a) Bray Curtis | | | (b) Jaccard | | |
| --- | --- | --- | --- | --- | --- | --- | --- |
| Source | df | MS | pseudo-*F* | *p* | MS | pseudo-*F* | *p* |
| Te | 1 | 4791 | 3.41 | **<0.001** | 6118 | 2.32 | **<0.001** |
| pH | 1 | 2695 | 1.92 | **0.004** | 3458 | 1.31 | **0.022** |
| Co | 1 | 11742 | 8.37 | **<0.001** | 6978 | 2.65 | **<0.001** |
| Te x pH | 1 | 2106 | 1.50 | **0.039** | 3051 | 1.16 | 0.153 |
| Te x Co | 1 | 4466 | 3.18 | **<0.001** | 4229 | 1.61 | **<0.001** |
| pH x Co | 1 | 3019 | 2.15 | **0.002** | 4020 | 1.53 | **0.005** |
| Te x pH x Co | 1 | 3859 | 2.75 | **<0.001** | 4803 | 1.82 | **<0.001** |
| Residual | 24 | 1403 |  |  | 2635 |  |  |
| Pairwise tests: | |  |  |  |  |  |  |
| Te x pH x Co, for Co | | 21^o^C Cu&Fu, 23.5^o^C Cu: H≠B  23.5^o^C Fu: H=B | | | 21^o^C Fu: H≠B  21^o^C Cu&Fu, 23.5^o^C Cu: H=B | | |
| Te x pH x Co, for Te | | Fu B: 21≠23.5^o^C  Cu H&B, Fu H: 21=23.5^o^C | | | Fu B: 21≠23.5^o^C  Cu H&B, Fu H: 21=23.5^o^C | | |
| Te x pH x Co, for pH | | 23.5^o^C B: Cu≠Fu  21^o^C H&B, 23.5^o^C H: Cu=Fu | | | All groups: Cu=Fu | | |


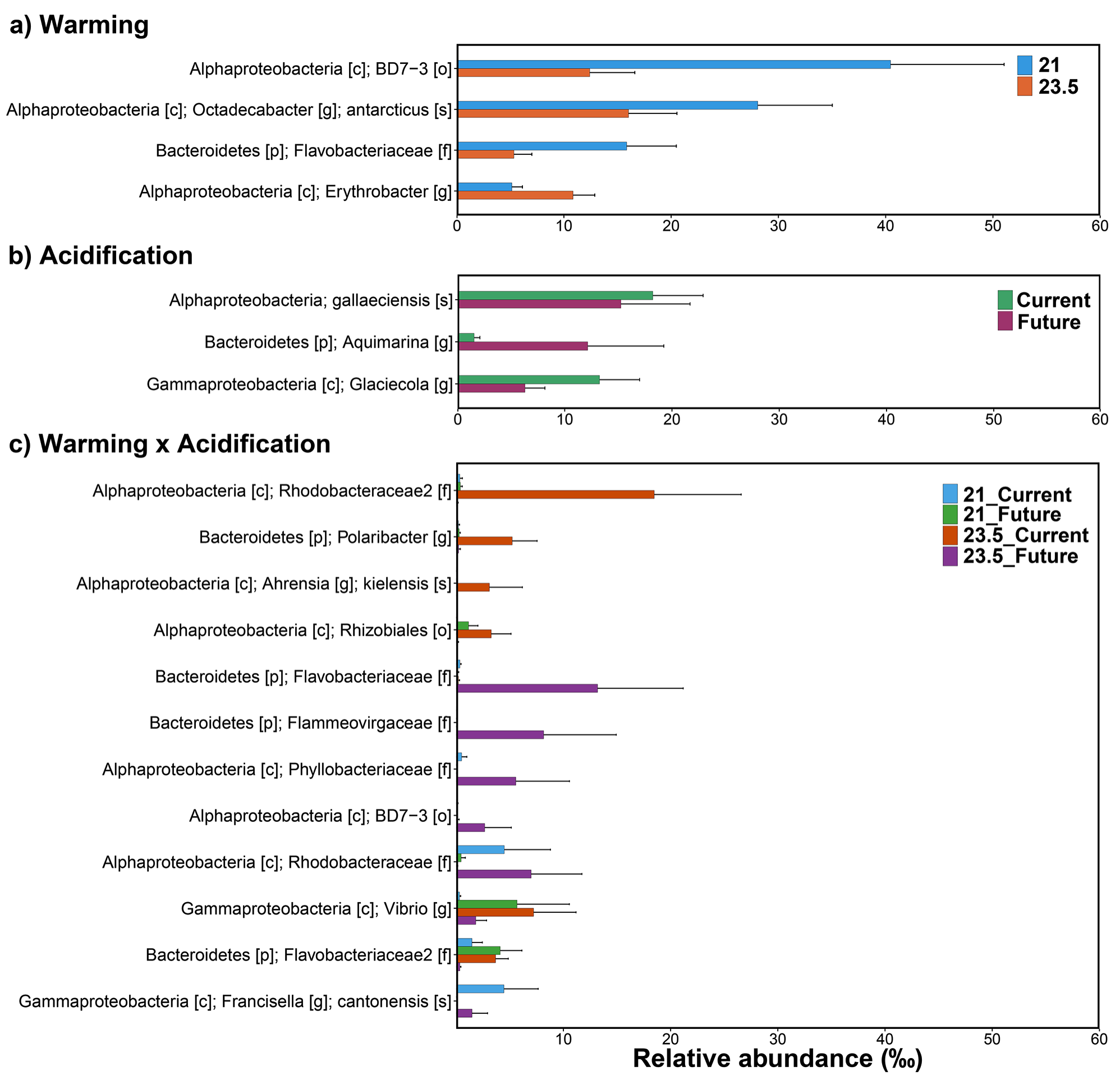


**Fig. S2.** OTUs affected significantly by warming and acidification. Mean relative abundances (+SE, n=10-12) of OTUs found to differ strongly between temperature (a; 21 vs 23.5^o^C) and pH treatments (b; ‘Current’ vs ‘Future’), or their interaction (c) ([p] = Phyllum, [c] = Class, [o] = Order, [f] = Family, [g] = Genus, [s] = species).

**
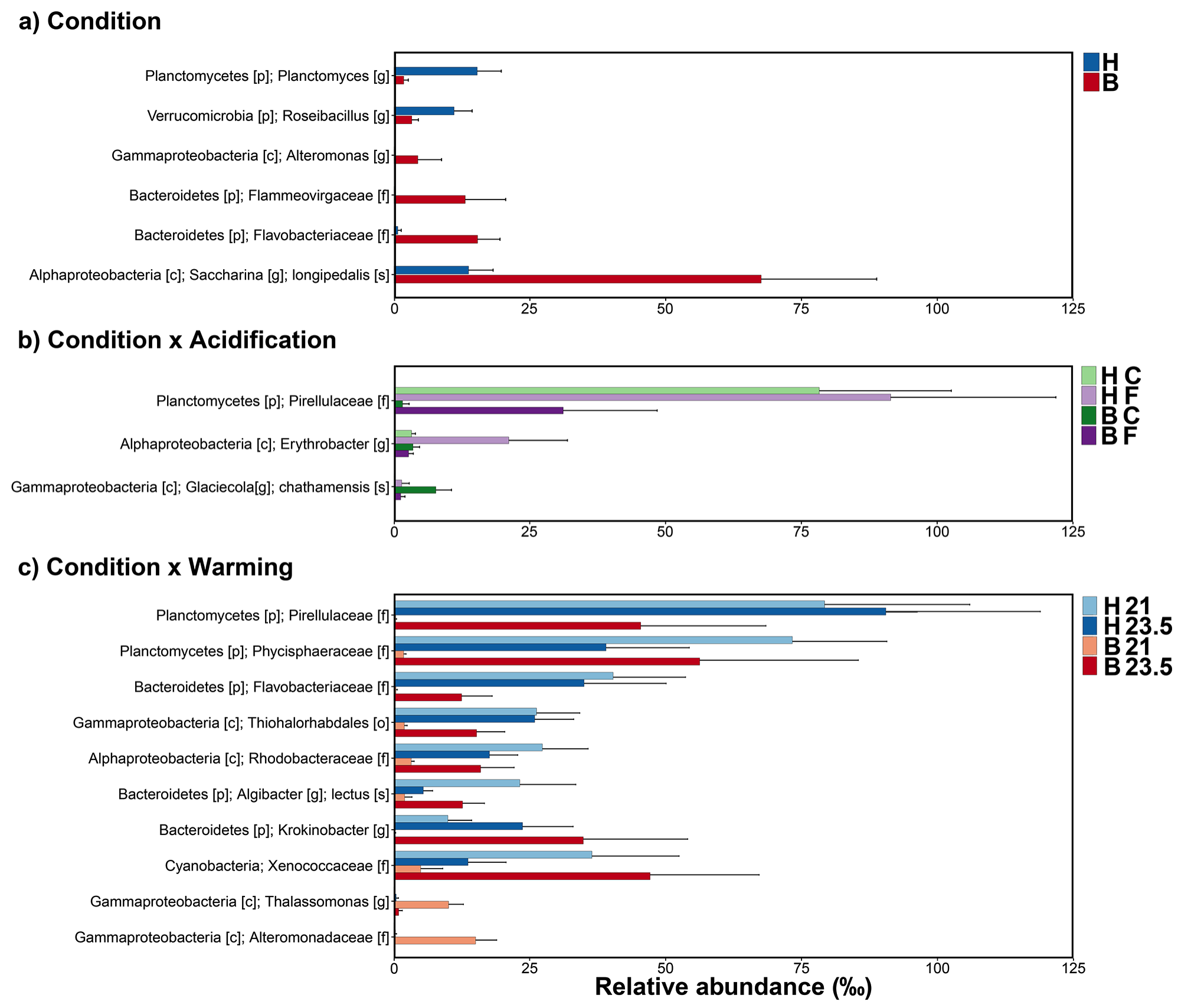
**

**Fig. S3.** OTUs affected significantly by kelp condition, warming and acidification. Mean relative abundances (+SE, n=16) of OTUs found to differ strongly between healthy (H) and blistered (B) kelp condition across all temperature (21 vs 23.5^o^C) and pH (Current, ‘C’ vs Future, ‘F’) treatments at day 16. OTUs shown for strong main effects of condition (a), or interactive effects of condition and pH (b), and condition and temperature (c).
